# Construct validity of the General Health Questionnaire (GHQ-12) in patients with COVID-19 and its demographic and medical correlates

**DOI:** 10.3389/fpsyg.2023.1132154

**Published:** 2023-06-05

**Authors:** Mojtaba Habibi Asgarabad, Farnaz Etesam, Pardis Salehi Yegaei, Zahra Vahabi, Niusha Akbari Saneh, Fatemeh Fathi, Fatemeh Ghosi, Nora Wiium

**Affiliations:** ^1^Health Promotion Research Center, Iran University of Medical Science, Tehran, Iran; ^2^Department of Psychology, Norwegian University of Science and Technology, Trondheim, Norway; ^3^Department of Health Psychology, School of Behavioral Sciences and Mental Health, Tehran Psychiatric Institute, Iran University of Medical Sciences, Tehran, Iran; ^4^Positive Youth Development Lab, Human Development and Family Sciences, Texas Tech University, Lubbock, TX, United States; ^5^Center of Excellence in Cognitive Neuropsychology, Institute for Cognitive and Brain Sciences, Shahid Beheshti University, Tehran, Iran; ^6^Department of Geriatric Medicine, Tehran University of Medical Sciences, Ziaeian Hospital, Tehran, Iran; ^7^Psychosomatic Research Center, Department of Psychiatry, Tehran University of Medical Sciences, Imam Khomeini Hospital, Tehran, Iran; ^8^Cognitive Neurology and Neuropsychiatry Division, Department of Psychiatry, Tehran University of Medical Sciences, Roozbeh Hospital, Tehran, Iran; ^9^Department of Clinical Psychology, School of Behavioral Sciences and Mental Health, Tehran Psychiatric Institute, Iran University of Medical Sciences, Tehran, Iran; ^10^Faculty of Psychology and Educational Sciences, Semnan University, Semnan, Iran; ^11^Department of Psychology and Educational Sciences, University of Al Zahra, Tehran, Iran; ^12^Department of Psychosocial Science, Faculty of Psychology, University of Bergen, Bergen, Norway

**Keywords:** COVID-19, concurrent validity, general health, medical condition, reliability, sleep, activities of daily life, stress

## Abstract

**Introduction:**

The present cross sectional study aimed to evaluate the construct and criterion validity, reliability, and gender and age differences of the 12-item General Health Questionnaire (GHQ-12) among hospitalized patients with COVID-19 in 2020. The criterion validity was assessed *via* its link with perceived stress, sleep quality, daily life activities, and demographic and medical characteristics.

**Methods:**

A total of 328 COVID-19 patients (55.8% men; M_age_ = 50.49, SD = 14.96) completed the GHQ-12, the Perceived Stress Scale (PSS), the Pittsburgh Sleep Quality Index (PSQI), the Activities of Daily Life (ADL)-Katz Scale, and the Lawton Instrumental Activities of Daily Living Scale (IADL).

**Results:**

Among 13 factorial models, the three-factor model (successful coping, self-esteem, and stress) was shown to have the best fit. GHQ-12 was positively associated with PSQI, PSS, Hyperlipidemia, psychiatry disorders, hospitalization duration, the change in sleep time, and use of sleeping pills, and negatively correlated with educational level, and the number of family members. The GHQ-12 also had a negative correlation with ADL and IADL in over 60 years of age group. Females scored higher on total GHQ-12 scores, compared to males. Finally, the hospitalization duration was longer for patients over 60 (mean = 8.8 days, SD = 5.9) than patients under 60 (mean = 6.35 days, SD = 5.87).

**Discussion:**

Overall, the findings provided evidence that mental distress in patients with COVID-19 is correlated with high perceived stress, low sleep quality, low ADL and IADL, and a range of demographic features and medical conditions. Designing psychological interventions for these patients that target the aforementioned correlates of mental distress is warranted.

## Introduction

New measures of the COVID-19 pandemic (e.g., self-isolation and quarantine) have led to an increase in mental distress, such as anxiety, insomnia, and suicidal attempt (WHO, 2020; Santomauro et al., 2021). Review studies after the COVID-19 pandemic showed high prevalence rate of anxiety (27–41.3%) and depression (27 to 34.1%) in Eastern Europe (Zhang et al., 2022), Southeast Asia (Pappa et al., 2022) and South Asia (Hossain et al., 2021).

To capture changes in mental health in both general and clinical populations, the self-administered General Health Questionnaire (GHQ; Goldberg, 1988) was developed. The 12-item short-form of GHQ (GHQ-12; Goldberg et al., 1997) was derived from the original 60-item questionnaire for fast administration in busy clinical settings. This questionnaire screens those with common psychological problems, such as poor self-esteem, stress, and sleep loss (del Pilar Sánchez-López and Dresch, 2008).

Although GHQ-12 was originally designed as a unidimensional measure (Goldberg, 1972), several exploratory factor analyses indicated that a two-factor (Zhong et al., 2021) or three-factor model (del Pilar Sánchez-López and Dresch, 2008) is the most common model (full information of these factorial models and their reliabilities are presented in Table 1). However, considering the study of Liang et al. (2016) for instance, they failed to find the best fitting model among ten existing factorial models, highlighting that there is a need for further research on the factor structure of GHQ-12.

**Table 1:** Studies Validating the Psychometric Properties of the GHQ-12 in Different Populations.

Author	Country	Participant characteristics	Factor structure and fit indices	Factors and corresponding items	Reliability
**Unidimensional models**					
Alaminos-Torres et al. (2021)	Spain	*n* = 342 Age range = 41–50 years	Range of factor loadings (EFA; unidimensional model) = 0.57–0.95	–	Total score = 0.85
Gnambs and Staufenbiel (2018)	Germany	*n*_1_ = 76,473 *n*_2_ = 410,640	Fit index (CFA; unidimensional model) = CFI = 0.89, RMSEA = 0.11	–	Total score = 0.85
Hystad and Johnsen (2020)	Norway	*n*_1_ = 591 *n*_2_ = 196	Fit index (CFA; unidimensional model) = CFI = 0.91, RMSEA = 0.07	–	–
Romppel et al. (2013)	Germany	*n* = 2,041 (53% female) M_age_ (SD) = 48.8 (18.1)	Fit indexes (CFA; unidimensional model) = CFI = 0.93, RMSEA = 0.10	–	Total score = 0.89 Positively worded items = 0.79 Negatively worded items = 0.86
**Two-factor models**					
Hamad (2022)	Saudi Arabia	*n* = 473 (60.81% female)	Fit index (CFA; two-factor model) = CFI = 0.96, RMSEA = 0.05	F1 Personal and Social Dysfunction = 1,3,4,7,8,9,10,11,12 F2 Anxiety = 2,5,6	Total score = 0.85
Kalliath et al. (2004)	New Zealand	*n*_1_ = 691 (54% female) M_age_ = 38 *n*_2_ = 415 (54% female) *M*_age_ = 38	Fit index (CFA; two-factor model) = CFI = 0.98, RMSEA = 0.07	F1Social Dysfunction = 4,7,8,12 F2 Anxiety/ Depression = 6,9,10,11	Total score at T_1_ = 0.91 Total score at T_2_ = 0.90
Montazeri et al. (2003)	Iran	*n* = 748 (76% female) M_age_ (SD) = 21.1 (2.1)	Range of factor loadings (EFA; two-factor model) = F_1_ = 0.56–0.81; F_2_ = 0.46–0.69	F1 Psychological distress = 1,3,4,7,8,10,11 F2 social dysfunction = 2,5,6,7,9,12	Total score = 0.87
Najarkolaei et al. (2014)	Iran	*n* = 428 (56% female) M _age_ (SD) = 22.83 (3.09)	Range of factor loadings (EFA; two-factor model) = F_1_ = 0.46–0.73; F_2_ = 0.39–0.78 Fit indexes (CFA; two-factor model) = GFI = 0.96, RMSEA = 0.04	F1 social dysfunction = 1,2,5,7,9,12 F2 psychological distress = 3,4,6,8,10,11	Total score = 0.85 Social dysfunction = 0.77 Psychological distress = 0.76
Politi et al. (1994)	Italy	*n* = 320 (0% female) M _age_ (SD) = 18	Range of factor loadings (EFA; two-factor model) = F_1_ = 0.31–0.80; F_2_ = 0.34–0.72	F1 general dysphoria = 2,5,6,9,10,11,12 F2 social dysfunction = 1,3,4,7,8	Total score = 0.81
Schrnitz et al. (1999)	Germany	*n* = 572 (68.7% female) M _age_ (SD) = 42.7 (15.7)	A principal-components factor analysis = factors’ eigenvalues of >1	F1 Anxiety/ Depression = 1,2,6,7,10,11 F2 Social Performance = 4, 5, 8, 9 and 12	Anxiety/ Depression = 0.86 Social Performance = 0.82 Total score = 0.91
Zhong et al. (2021)	China	–	Range of factor loadings (EFA; two-factor model) = F_1_ = 0.62–0.72; F_2_ = 0.48–0.79 Fit indexes (CFA; two-factor model) = 0.92, RMSEA = 0.08	F1 = 1,2,5,7,9,12 F2 = 3,4,6,8,10,11	Total score = 0.89
**Three-factor models**					
Daradkeh et al. (2001)	United Arab Emirates	*n* = 157	Range of factor loadings (EFA; three-factor model) = F_1_ = 0.57–0.79; F_2_ = 0.44–0.80; F_3_ = 0.55–0.87	F1general dysphoria = 10, 5, 9, 11, 6 F2 lack of enjoyment = 7, 12, 1, 8, 2 F3 social dysfunction = 3, 4	Total score = 0.86
del Pilar Sánchez-López and Dresch (2008)	Spain	*n* = 1,001 (60% female) M_age_ (SD) = 41.75 (10.95)	Range of factor loadings (EFA; three-factor model) = F_1_ = 0.50–0.71; F_2_ = 0.41–0.63; F_3_ = 0.63–0.65	F1Successful Coping = 1,3,4,7,8,12 F2 Self-esteem = 6,9,10,11 F3 Stress = 2,5,9	Total score = 0.76
Farrell (1998)	Australia	*n* = 270 (85% female) M_age_ (SD) = 0.36 (9)	Range of factor loadings (EFA; three-factor model) = F_1_ = 0.62–0.81; F_2_ = 0.60–0.80; F_3_ = 0.70–0.84	F1 Anxiety = 10,12,2,5,11 F2 Depression = 1,9,8,7,6 F3 Social dysfunction = 3,4	Anxiety = 0.84 Depression = 0.81 Social dysfunction = 0.69
Gao et al. (2004)	Singapore	*n* = 120 (47.5% female) M_age_ (SD) = 43.1 (12.7)	Fit index (CFA; three-factor model model) = CFI = 0.93, RMSEA = 0.10	F1 Anxiety and depression = 2,5,9,6, F2 Social dysfunction = 1,3,4,8,7,12 F3 Loss of confidence = 10,11	–
Graetz (1991)	Australia	n = 8,998 (49% female) Age range = 16–25 years	Range of factor loadings (EFA; three-factor model) at T_1_ and T_2_ = F_1_ = 0.44–0.78; F_2_ = 0.44–0.59; F_3_ = −0.70 – -0.72 Range of factor loadings (EFA; three-factor model) at T_3_ and T_4_ = F_1_ = 0.38–0.77; F_2_ = 0.44–0.64; F_3_ = −0.72 – -0.74	F1 Anxiety and depression = 2,5,6,9 F2 Social dysfunction = 1,3,4,7,8,12 F3 loss of confidence = 10,11	–
Lee and Kim (2020)	South Korea	*n* = 504 (66.8% female) M_age_ (SD) = 20.2 (1.63)	Fit indexes (CFA; three-factor model) = CFI = 0.93, RMSEA = 0.07	F1 Anxiety and depression = 2,5,6,9 F2 Social dysfunction = 1,3,4,7,8,12 F3 loss of confidence = 10,11	Total score = 0.81
Liang et al. (2016)	China	*n* = 1,051 (38.5% female) Age range = 29–35 years	Three-dimensional model (CFA) = CFI = 0.98, RMSEA = 0.03	F1 = 4,6,9,10,11,12 F2 = 3,5,7,8 F3 = 1,2	Total score = 0.84
Martin (1999)	Australia	*n* = 169 (61.1% female) M _age_ (SD) = 28 (11)	Range of factor loadings (EFA; three-factor model) = F_1_ = 0.46–0.64; F_2_ = 0.65–0.70; F_3_ = −0.63–0.84	F1 Self-esteem = 1,3,4,8 F2 Stress = 2,5,7 F3 Successful Coping = 6,9,10,11,12	Self-esteem = 0.83 Stress = 0.71 Successful Coping = 0.67

*n*, sample size; *F*, factor; *T*, study wave; *M*, mean; CFI, comparative fit index; RMSEA, root mean square error of approximation; GFI, goodness of fit index; EFA, exploratory factor analysis; CFA, confirmatory factor analysis. Inconsistency in reporting the demographic characteristics including sample size, percentage of females, Mean_age_ (*SD*) in column “participants” is due to not reporting the relevant data in the papers.

### General mental health: Stress and sleep quality

Mental health is reciprocally linked to sleep quality, specifically in patients with COVID-19 who are commonly susceptible to sleep disturbances (Deng et al., 2021; Marvaldi et al., 2021). Perceived stress, referring to the extent to which a person perceives their daily life situations as stressful, was also found to be positively associated with GHQ-12 in a large cross-national sample of COVID-19 patients (Bonsaksen et al., 2022). Patients infected with COVID-19 experienced the burden of job loss (Crayne, 2020), death anxiety (Korkut, 2022), and may react with heightened stress (Bonsaksen et al., 2022).

### General mental health: Medical conditions and demographic characteristics

Coronavirus may severely impair the subsequent physical functioning in some patients, especially the elderly (Carfì et al., 2020; Halpin et al., 2020). The Activities of daily living (ADL) and the Instrumental Activities of Daily Living (IADL; Katz et al., 1970; Lawton, 2000) are basic skills necessary for independently taking care of oneself (Edemekong et al., 2017) and environmental adaptation (Roehrig et al., 2007). Although few in number, some studies have shown the link of mental health to ADL and IADL scores (de Castroe Costa et al., 2008).

Due to the negative effect of COVID-19 on several aspects of people’s lives (Santomauro et al., 2021), research should document the association of mental health status in COVID-19 patients with demographics and medical features. Previous studies have suggested the positive link of mental health status with medical features, such as change in sleep time before and after COVID-19 and use of sleeping pills (Becker et al., 2018), hospitalization duration (Liao et al., 2020), psychiatry disorders (Kaufman et al., 2020), hyperlipidemia (HLP; Chang et al., 2021), diabetes (Moradian et al., 2021), cardiovascular disease (CVD; de Paiva Teixeira et al., 2020), substance use history (Czeisler et al., 2020), as well as demographics characteristics, namely, educational level (Dalgard et al., 2007), unemployment (Achdut and Refaeli, 2020), and the number of family members (Hendriksen et al., 2021). COVID-19 pandemic was shown to result in greater mental distress in women (Giorgi et al., 2014; Bucciarelli et al., 2021). As an example, in a study on large data of 49,156 participants, Proto and Quintana-Domeque (2021) found that after the COVID-19 pandemic, women manifested higher elevation in GHQ-12 scores (higher psychological distress) than men. Aging is believed to be associated with decrease in mental distress (Hoeymans et al., 2004), while COVID-19 pandemic have led to greater mental distress in younger patients (Bruine de Bruin, 2021).

### The study context

Iran is among the worst-hit countries by Coronavirus, with heavy death tolls (more than 19,000 deaths until August 2020; Shahriarirad et al., 2021). Challenging factors, namely, the shortage of hygiene and medical supplies and equipment (i.e., masks and disinfectants), economic constraints (Zandifar and Badrfam, 2020), and the incapacity of government to formulate and enforce effectual social distancing and lockdown measures have led to the mental distress in the Iranian general population (Moghanibashi-Mansourieh, 2020; Zandifar and Badrfam, 2020; Shahriarirad et al., 2021). The Iranian population has been no exception to the global trend of increased mental issues. In a group of 5,328 individuals from the general population of Iran, the prevalence rates of anxiety, depression, and comorbid depression-anxiety were determined to be 30.1, 33.4, and 22.1%, respectively (Nakhostin-Ansari et al., 2020). Moreover, in another recent study by Maroufizadeh et al. (2022), the prevalence of mild-to-severe anxiety and depression in Iranian medical students was found to be 38.1 and 27.6%, showing a significant impact on sleep patterns.

In Iran, two studies have assessed the psychometric properties of GHQ-12. In a study on emerging adults, Montazeri et al. (2003) findings confirmed the two-factor model, comprising “depression” and “social dysfunction.” Their study showed the negative association of GHQ-12 with global quality of life, supporting its satisfactory convergent validity. Similarly, the results of Najarkolaei et al. (2014) study supported a two-factor model including “distress” and “social dysfunction” in freshmen university students. Nevertheless, participants of these two studies had limited age range (18–26 years of age) and were recruited from university students, which prevent their results from being generalized to the general or clinical Iranian population.

The present study was first-of-its-kind that aimed to examine the psychometric properties of the GHQ-12 in Iranian patients with COVID-19. In specific, we aimed to examine: (1) the factor structure by conducting Confirmatory Factor Analysis (CFA) based on 13 empirically-derived factorial models, (2) the internal consistency, (3) the criterion validity through the relationship of GHQ with perceived stress, sleep quality, ADL/IADL, and demographic and medical variables, and (4) the comparison of the average GHQ-12 scores among age and gender groups (if any). We hypothesized that higher GHQ-12 score—that reflects lower mental health—has a positive relationship with: (1) poor sleep quality, (2) higher perceived stress, and (3) lower level of ADL/IADL functions.

## Materials and methods

### Participants

Participants comprised a total of 328 patients with COVID-19 (55.8% men), aged 21 to 92 (Mean_age_ (SD) = 50.49 (14.96); 73.6% 60 years old or younger). As for educational level, 19.8% of participants were illiterate, 22.9% had primary education, 16.5% had secondary education, 23.5% had diploma level, and 17.3% had higher education. Their job status included 15.3% employee, 6.7% skill-worker, 20.7% self-employed, 39.6% unemployed, and 13.1% retired. Most patients (88.6%) were living with their spouse and/or their children, while 10.5% were living alone. A majority of 86.6% had no history of smoking, while 10.1 and 2.4% reported smoking in the past and at the present time. In addition, 93.9 and 96.6% reported no history of alcohol and drug use, respectively. Among participants, 4.6% reported using sleeping pills—mostly Alprazolam, Chlordiazepoxide, and Asentra. Patients under and over 60 reported underlying diseases, including Diabetes (13 and 44.6%), High Blood Pressure (HTN; 15.2 and 28.9%), HLP (11.7 and 31.3%), psychiatry disorders (4.8 and 3.6%), immune deficiency disease (IDD; 1.3 and 4.8%), and Cardiovascular disease (9.1 and 28.9%), each. Finally, the mean of hospitalization duration was 6.35 days (SD = 5.87) for patients under 60 and 8.8 days (SD = 5.9) for patients over 60.

### Measurements

#### General Health Questionnaire

The self-report GHQ-12 was developed to screen global mental state (Goldberg et al., 1997). Among two common scoring methods of the bi-modal (*0–0–1-1*) and *Likert* scoring (*0–1–2-3*) types, the *Likert* method is preferable since it measures the symptom severity on a continuum (Hystad and Johnsen, 2020). In this study, the scoring based on the 4-point *Likert*-scale (*0–1–2-3*) was used, in which: 0 = “*not at all*,” 1 = “*no more than usual*,” 2 = “*rather more than usual*,” and 3 = “*much more than usual*,” where a higher score indicated lower mental health (Goldberg et al., 1997).

#### Pittsburgh Sleep Quality Index

This 19-item self-administered tool (Buysse et al., 1989) was designed for brief assessment of seven components: (1) subjective sleep quality (e.g., “*how would you rate your sleep quality overall?*”), (2) sleep latency (e.g., “*cannot get to sleep within 30 min*”), (3) sleep duration (e.g., “*how many hours of actual sleep do you get at night?*”), (4) sleep efficiency (e.g., “*when have you usually gone to bed?*”), (5) sleep disturbances (e.g., “*wake up in the middle of the night or early morning*”), (6) use of sleeping medication (e.g., “*how often have you taken medicine to help you sleep?*”), and (7) daytime dysfunction (e.g., “*how often have you had trouble staying awake while driving,…*”). Each component was weighted on a *Likert* scale from 0 to 3, with higher scores indicating poorer sleep quality. *Cronbach*’s alpha in the current study was.77 for the total score. The Persian version of PSQI that showed adequate psychometric properties (Farrahi Moghaddam et al., 2012) was used in the current study.

#### Perceived Stress Scale

PSS is a 10-item unidimensional scale (Cohen et al., 1983) that measures how much patients appraise the situations in their life as stressful during the preceding month. Items were coded based on a 5-point *Likert*-type scale: 0 (*never*), 1 (*almost never*), 2 (*once in a while*), 3 (*often*), and 4 (*very often*). Higher scores indicated higher perceived stress (e.g., *“unable to control the important things in your life”*). *Cronbach*’s *α* for the Persian version of PSS was.84 for the total score (Maroufizadeh et al., 2018), while an alpha value of.68 was obtained in our study.

#### ADL-Katz Scale and The Lawton Instrumental Activities of Daily Living Scale

The 6-item ADL-Katz Scale (Katz et al., 1970) assessed the ability of bathing, transferring, dressing and grooming, walking, toileting, and feeding in people over 60 years of age. The Lawton Instrumental Activities of Daily Living Scale (Lawton, 2000) measured instrumental functioning, namely, using the phone, doing housework, doing laundry, managing transportation, shopping, cooking, managing medications, and managing finances. Items for both ADL and IADL were scored based on 0 (*no*) and 1 (*yes*). *Cronbach*’s alphas for the Persian versions of ADL and IADL were 0.80 (Sharifi et al., 2018) and between 0.72 and 0.76 (Mehraban et al., 2014), respectively. In this study, alphas were 0.66 and 0.82 for ADL and IADL, respectively.

#### Medical conditions and demographic characteristics

In order to evaluate the patients’ demographics and medical characteristics, a questionnaire constructed by researchers was used. The medical features included: (a) the history of underlying diseases (Diabetes, HTN, HLP, Myocardial Infarction (MI), Cerebrovascular Accident (CVA), Pulmonary Disease (PD), Kidney failure, Psychiatry disorders, Obesity, IDD, and CVD) (*yes/no*), (b) cigarette, alcohol, and drug history (*yes/no*), (c) hospitalization duration (*days*), (d) use of downer or sleeping pills (*yes/no*), and (e) the change in sleep time before and after COVID-19 (*hours*). The demographic characteristics included: (a) gender (*male/female*), (b) age (*years*), (c) job status (*employed/unemployed*), (d) educational level (*illiterate, primary education, secondary education, diploma level, and higher education*), and (e) the number of family members.

### Procedure

The current cross-sectional study was carried out on patients hospitalized due to Coronavirus infection at the Baharloo and Ziaeian Hospitals from March to October 2020 in Tehran, Iran. After being discharged from the hospital, those who accepted to take part in the current study were asked to sign the consent form. The demographic information of those who consented to take part and their contact number was collected in a registration form. Then, three psychologists collected the data (demographic and medical variables, GHQ-12, PSS-10, PSQI, ADL and IADL), using telephone survey. Patients were informed about their optional participation in the current research and that they can leave the research any time they wish. This study received ethic approval from the Review Board of Tehran University of Medical Sciences (Ethical Code: IR.TUMS.VCR.REC.1399.156).

### Statistical strategy

Data screening was performed *via* IBM SPSS Statistics (Version 28). CFA tests of the GHQ-12 were conducted using Mplus version 8.8. Evaluating the assumption of normality revealed a mostly positive but non-substantial skewness in all items; thus, transformation was not required (Gravetter et al., 2020). We applied CFA using the Weighted Least Square Mean and Variance Adjusted (WLSMV) estimator. Statistical strategies were as follows: First, we used the following statistical tests and indices (MacCallum et al., 1996; Hu and Bentler, 1999; Hooper and Coughlan, 2008) to assess the models’ “goodness-of-fit” (acceptable values in parenthesis): the Chi-square (*χ^2^*; desired *p* > 0.05), the Comparative Fit Index (CFI > 0.95), the Tucker–Lewis Index (TLI > 0.95), the Standardized Root Mean Square Residual (SRMR <0.08), the Normal Chi-square (*χ^2^*/df < 5), the Root Mean Square Error of Approximation (RMSEA <0.10), and its 90% Confidence Interval (Bentler and Bonett, 1980; MacCallum et al., 1996; Loehlin, 2004; Miles and Shevlin, 2007). The exact fit is defensible when the Chi-square is not significant, regardless of the SRMR value. Approximate fit is tenable when Chi-square is significant, SRMR ≤0.08, and standard residuals are all small (|r_res_| < 0.1), and finally poor fit is concluded if Chi-square is significant, and SRMR >0.08 (Satorra and Bentler, 2010).

Second, for internal consistency—as recommended for ordinal *Likert*-type scales, the equivalents of *Cronbach*’s alpha coefficient (Ordinal *Theta* and *Omega* reliability coefficients) using R version 4.1.2 (Team, R. C, 2013; Revelle, 2017) were conducted, which instead of the Pearson correlation matrix, apply the poly-choric correlation matrix (Zumbo et al., 2007; Gadermann et al., 2012). A reliability coefficient of 0.70 or higher was considered an acceptable level (Cicchetti, 1994).

Third, the criterion validity was evaluated by the Spearman coefficient of rank correlation of GHQ-12 with PSQI, PSS-10, and ADL/IADL, since the data showed evidence of non-normality. Correlation coefficients were interpreted based on the effect size classification of Cohen (1988): 0.10 = small, 0.30 = medium, 0.50 = large, and 0.70 = very large.

Forth, Multivariate Analysis of Variance (MANOVA) and effect size (Hedge’s g) were used to compare the mean and standard deviation of the GHQ-12 scores across gender. According to a rule of thumb suggested by Cohen (1988), effect sizes were classified into small (<0.20), medium (0.21–0.50), large (0.51–0.80), and very large (>0.80).

## Results

### Aim 1: GHQ-12 construct validity

To test the GHQ-12 factor structure, CFA was conducted and the goodness of fit for 13 models was examined (Table 2). Model 1 (M_1_) examined a general factor, in which, the total of the 12 items were loaded on a single common factor of general mental health (Goldberg et al., 1997; Romppel et al., 2013; Gnambs and Staufenbiel, 2018; Hystad and Johnsen, 2020; Alaminos-Torres et al., 2021) to test the unidimensional model of assumed latent factor and included just random measurement error and indicator-specific variance (Gustafsson and Åberg-Bengtsson, 2010). If the general factor model fitted the data well, it meant that the assumption of the multidimensionality of the measurement tool was violated. Models two to seven (M_2_ to M_7_) consisted of a the first-order two-factor oblique models that suggested two subscales measuring two distinct dimensions (Politi et al., 1994; Schrnitz et al., 1999; Montazeri et al., 2003; Kalliath et al., 2004; Najarkolaei et al., 2014; Zhong et al., 2021; Hamad, 2022). Models 8–13 (M_8_ to M_13_) examined first-order three-factor oblique models, resembling the Exploratory Factor Analysis (EFA) according to the literature (Graetz, 1991; Farrell, 1998; Martin, 1999; Daradkeh et al., 2001; Gao et al., 2004; del Pilar Sánchez-López and Dresch, 2008; Liang et al., 2016; Lee and Kim, 2020). Model 8 (M_8_) included general dysphoria, lack of enjoyment, and social dysfunction (Daradkeh et al., 2001). Model 9 (M_9_) consisted of anxiety, depression, and social dysfunction (Farrell, 1998). Model 10 (M_10_) was loaded by all three first-order factors, which included social dysfunction, anxiety and depression, and loss of confidence (Graetz, 1991; Gao et al., 2004; Lee and Kim, 2020). Model 11 (M_11_) included cope, stress, and low self-esteem (Martin, 1999). Model 12 (M_12_) comprised low level of social function, anxiety/depression, and poor self-confidence (Liang et al., 2016). Finally, Model 13 (M_13_) included successful coping, self-esteem, and stress (del Pilar Sánchez-López and Dresch, 2008).

**Table 2:** Fit indices of the Measurement Models of the GHQ-12.

Model	*χ^2^*	*df*	*χ^2^*/df	CFI	TLI	RMSEA	SRMR	Based Model	Δ*χ*^2^ (df)
M_1_ (Goldberg et al., 1997; Romppel et al., 2013; Gnambs and Staufenbiel, 2018; Hystad and Johnsen, 2020; Alaminos-Torres et al., 2021)	1030.608	54	19.08	0.900	0.878	0.240 (0.227–0.253)	0.172	–	–
M_2_ (Politi et al., 1994)^†^	1016.411	53	19.17	0.902	0.878	0.241 (0.228–0.254)	0.170	M_1_	–
M_3_ (Kalliath et al., 2004)^†^	458.688	19	24.14	0.869	0.806	0.271 (0.250–0.293)	0.099	M_1_	–
M_4_ (Montazeri et al., 2003)^†^	955.448	52	18.37	0.908	0.883	0.235 (0.222–0.248)	0.165	M_1_	–
M_5_(Najarkolaei et al., 2014; Zhong et al., 2021)^†^	1023.869	53	19.31	0.901	0.877	0.242 (0.229–0.255)	0.170	M_1_	–
M_6_ (Schrnitz et al., 1999)^†^	951.700	43	22.13	0.825	0.776	0.259 (0.245–0.274)	0.134	M_1_	–
M_7_ (Hamad, 2022)^†^	1017.709	53	19.20	0.902	0.878	0.241 (0.228–0.254)	0.172	M_1_	–
M_8_ (Daradkeh et al., 2001)	672.991	51	13.19	0.938	0.920	0.197 (0.184–0.210)	0.099	M_1_	357.62 (3)^***^
M_9_ (Farrell, 1998)^†^	1016.582	51	19.93	0.904	0.876	0.246 (0.233–0.259)	0.135	M_1_	–
M_10_ (Graetz, 1991; Gao et al., 2004; Lee and Kim, 2020)	530.411	51	10.40	0.952	0.938	0.173 (0.160–0.187)	0.087	M_1_	500.20 (3)^***^
M_11_ (Martin, 1999)	751.738	51	14.73	0.930	0.910	0.209 (0.196–0.223)	0.107	M_1_	278.87 (3)^***^
M_12_ (Liang et al., 2016)^†^	3018.809	50	60.37	0.704	0.610	0.435 (0.422–0.448)	0.287	M_1_	–
M_13_ (del Pilar Sánchez-López and Dresch, 2008)††	**502.118**	**50**	**10.04**	**0.955**	**0.941**	**0.170 (0.156–0.183)**	**0.080**	**M**_**1**_	528.49 (4)^***^

*χ*^2^, Chi-square; df, degrees of freedom; TLI, Tucker–Lewis index; CFI, comparative fit index; ABIC, sample-size adjusted Bayesian information criterion; χ^2^/df, normal Chi-square; Δχ^2^, difference between minus twice log likelihoods between the full and the nested models; SRMR, standardized root mean square residual; RMSEA, root mean square error of approximation; Δ, differences between parameters of two models; † = The problem with the model is that the factors correlate greater than one which makes the model inadmissible, then, model cannot be used. †† = The final selected model. ^∗^*p* < 0.05, ^∗∗^*p* < 0.01, ^∗∗∗^*p* < 0.001.The bold values correspond to the final selected model.

#### Model selection

In Table 2, all two-factor models (Politi et al., 1994; Schrnitz et al., 1999; Montazeri et al., 2003; Kalliath et al., 2004; Najarkolaei et al., 2014; Zhong et al., 2021; Hamad, 2022) are inadmissible, due to factors correlate greater than 1.00 between two latent factors. The correlation between the two latent factors in two out of six models for the three-factor model also exceeded 1.00, as shown in Table 2 (M_9_ and M_12_; Farrell, 1998; Liang et al., 2016). The fit indices of the three-factor oblique model for remaining four models (Table 2; M_8_, M_10_, M_11_, and M_13_) met some of the specified fit criteria, as prior, and based on the theory-derived models (Graetz, 1991; Martin, 1999; Daradkeh et al., 2001; Gao et al., 2004; del Pilar Sánchez-López and Dresch, 2008; Lee and Kim, 2020). Then, the parsimonious principle (Bollen, 1989) was used to compare the fit indices of the three-factor first-order oblique models (Table 2; M_8_ [Δ*χ^2^* = 357.62, Δdf = 3, *p* < 0.001], M_10_[Δ*χ^2^* = 500.20, Δdf = 3, *p* < 0.001], M_11_[Δ*χ^2^* = 278.87, Δdf = 7, *p* < 0.001], and M_13_[Δ*χ^2^* = 528.49, Δdf = 4, *p* < 0.001]) with those of the unidimensional first-order model (M_1_) as the baseline/null model. For three-factor models, four out of six models exhibited similar fitness, though with poor goodness-of-fit (Table 2). To determine the most efficient parsimonious model, the nesting and equivalence testing (NET) methodology was implemented *via* Mplus 8.8 (Bentler and Satorra, 2010; Asparouhov and Muthén, 2019). Since all models are non-tested and/or non-equivalent, as was expected (the NET value = 0.0000001), it can be concluded that model 13 showed the best fit, due to its low Chi-square value in comparison with the others (*χ^2^*/df = 10.04; CFI = 0.96; TLI = 0.94; RMSEA = 0.17; 90% CI = 0.16 to 0.18; SRMR = 0.08). More information on confirmatory factor analysis of models 1, 8, 10, 11, and 13 is presented in supporting information file (Supplementary Figures S1–S5).

### Aim 2: GHQ-12 reliability

The Ordinal *Theta* and the *Omega* reliability coefficients for the subscales of GHQ-12 are presented in Table 3. The means of inter-item correlation were 0.10, 0.53, 0.48, and 0.58 for the total score, successful coping, self-esteem, and stress, respectively. Almost all items within the three subscales had a moderate positive relationship with each other (based on the corrected item-total correlation for subscale’s items), with values ranging from 0.34 to 0.83, 0.23 to 0.78., and 0.53 to 0.66 for successful coping, self-esteem, and stress, respectively.

**Table 3:** The Descriptive statistics of GHQ-12.

	Mean (SD)					Ordinal *Theta*	*Omega*	*α*
	Total	Female	Male	Under 60	Over 60			
1. Successful Coping	8.95 (3.42)	8.39 (3.20)	9.39 (3.55)	9.30 (3.31)	7.98 (3.55)	0.90	0.86	0.87
2. Self-esteem	3.91 (2.63)	4.37 (2.61)	3.22 (2.39)	3.86 (2.54)	4.04 (2.88)	0.75	0.77	0.77
3. Stress	3.96 (2.43)	4.72 (2.40)	3.33 (2.27)	4.06 (2.40)	3.67 (2.51)	0.93	0.88	0.80
4. GHQ-12	15.52 (6.19)	16.18 (3.66)	14.87 (3.82)	15.88 (3.80)	14.39 (3.82)	-	-	0.86

*Notes*. GHQ = General health questionnaire, α = Alpha, SD = Standard deviation. ^∗^*p* < 0.05, ^∗∗^*p* < 0.01.

### Aim 3: GHQ-12 and related measures

Table 4 demonstrates that the inter-correlation between GHQ-12 total score and subscales ranged from 0.40 to 0.83 (*p* < 0.01). Criterion validity was estimated by the correlation of GHQ-12 total score and its subscales with PSQI and PSS (Table 4). PSQI had significant positive correlations with total GHQ-12 (*r* = 0.28, *p* < 0.01), successful coping (*r* = 0.24, *p* < 0.01), self-esteem (*r* = 0.21, *p* < 0.01), and stress (*r* = 0.20, *p* < 0.01). Also, PSS had significant positive correlations with the total score of GHQ-12 and its subscales (*r* = 0.31 to 0.58, *p* < 0.01). In the over 60 years of age group, negative correlations of the total GHQ-12 score were found with the ADL (*r* = −0.34, *p* < 0.01; *r* = −0.37, *p* < 0.01) and IADL scores (*r* = −0.42, *p* < 0.01, *r* = −0.46, *p* < 0.01) before and after the infection of COVID-19, respectively (Table 4).

**Table 4:** The correlation between GHQ-12 subscales and their correlations with sleep quality, perceived stress, and daily functioning.

	2	3	4	PSQI								PSS	ADL		IADL	
				Total	SSQ	SL	SD	SE	SD	USM	DD		Before	After	Before	After
1. Successful Coping	0.40^**^	0.45^**^	0.83^**^	0.24^**^	0.41^**^	0.24^**^	0.15	0.26^**^	0.29^**^	0.25^**^	0.51^**^	0.31^**^	-0.35^**^	-0.43^**^	-0.48^**^	-0.55^**^
2. Self-esteem		0.73^**^	0.80^**^	0.21^**^	0.43^**^	0.27^**^	0.20^*^	0.22^*^	0.27^**^	0.23^**^	0.23^**^	0.50^**^	-0.25^*^	-0.33^*^	-0.28^**^	-0.31^**^
3. Stress			0.81^**^	0.20^**^	0.57^**^	0.23^**^	0.38^*^	0.27^**^	0.48^**^	0.23^**^	0.28^**^	0.58^**^	-0.22^*^	-0.16	-0.19	-0.15
4. GHQ-12				0.28^**^	0.55^**^	0.30^**^	0.28^**^	0.30^**^	0.42^**^	0.30^**^	0.45^**^	0.53^**^	-0.34^**^	-0.37^**^	-0.42^**^	-0.46^**^

*Notes*. GHQ = General health questionnaire, PSQI = Pittsburgh sleep quality index, SSQ = Subjective sleep quality, SL = Sleep latency, SD = Sleep duration, SE = Sleep efficiency, SD = Sleep disturbances, USM = Use of sleeping medication, DD = Daytime dysfunction; PSS = Perceived stress scale. ^∗^*p* < 0.05, ^∗∗^*p* < 0.01.

As depicted in Table 5 for demographic and medical variables, the total score of GHQ-12 was significantly correlated with HLP (*r* = 0.16, *p* < 0.01), kidney failure (*r* = 0.12, *p* < 0.05), psychiatry disorders (*r* = 0.18, *p* < 0.01), the hospitalization duration (*r* = 0.15, *p* < 0.01), the change in sleep time (*r* = 0.31, *p* < 0.01), use of sleeping pills (*r* = 0.30, *p* < 0.01), educational level (*r* = −0.26, *p* < 0.01), and the number of family members (*r* = −0.12, *p* < 0.05). The correlation of the total score of GHQ-12 with Diabetes, HTN, MI, CVA, PD, obesity, IDD, CVD, and drug, alcohol, and cigarette history were non-significant (*p* > 0.01). Information for the correlation of GHQ-12 subscales is presented in Table 5.

**Table 5:** The correlations of Mental Health with demographic and medical variables.

	HLP	KF	Psychiatry disorders	Hospitalization duration	Change in sleep time	Use of sleeping pills	Educational level	Family members (n)
1. Successful Coping	0.14^*^	0.09	0.13^*^	0.17^**^	0.10	0.24^**^	-0.30^**^	-0.12^*^
2. Self-esteem	0.15^**^	0.08	0.14^*^	0.10	0.29^**^	0.25^**^	-0.17^**^	-0.11^*^
3. Stress	0.06	0.09	0.17^**^	0.08	0.44^**^	0.25^**^	-0.08	-0.05
4. GHQ-12	0.16^**^	0.12^*^	0.18^**^	0.15^**^	0.31^**^	0.30^**^	-0.26^**^	-0.12^*^

*Notes*. GHQ = General health questionnaire, HLP = Hyperlipidemia, KF = Kidney failure. ^∗^*p* < 0.05, ^∗∗^*p* < 0.01.

### Aim 4: Gender, age, and GHQ-12

Table 3 presents the mean and Standard Deviations (SD) of the GHQ-12 total score and subscales across gender and age groups. The female patients scored significantly higher than the males on total GHQ-12 scores [*t* (310) = −4.77, *p* < 0.001, Cohen’s *d* = −0.65, mean difference bootstrap 95% CI = −4.46 to-1.92]. The patients above 60 also scored slightly higher than patents under 60 on their total GHQ-12 scores [*t* (312) = −1.45, *p* = 0.08, Cohen’s *d* = −0.19, mean difference bootstrap 95% CI = −2.94 to 0.55]. A Multivariate Analysis of Variance (MANOVA) showed significant group differences by gender [*F* (3–308) = 10.80, *p* < 0.001, *η^2^* = 0.095] and age groups [*F* (3–310) = 7.27, *p* < 0.001, *η^2^* = 0.066] on mean scores of the subscales (see Table 3 for Mean scores). Subsequent tests of between-subjects’ effects showed that females scored significantly higher on successful coping [*F* (1–310) = 5.68, *p* < 0.05, *η^2^* = 0.018], self-esteem [*F* (1–310) = 28.11, *p* < 0.001, *η^2^* = 0.083], and stress [*F* (1–310) = 27.38, *p* < 0.081, η^2^ = 0.081] than males. The patients over 60 scored significantly higher on successful coping [*F* (1–312) = 9.41, *p* < 0.01, ns, η*^2^* = 0.029], than patients under 60. However, non-significant differences in mean score were found for self-esteem [*F* (1–312) = 0.31, *p* = 0.58, ns, *η^2^* = 0.001] = and stress [*F* (1–312) = 1.60, *p* = 0.21, ns, *η^2^* = 0.005] across age.

## Discussion

The present study aimed to assess the psychometric properties of the General Health Questionnaire-12 in patients hospitalized with a diagnosis of COVID-19. Overall, our results offer support for the construct validity, criterion validity, and internal consistency of GHQ-12. Therefore, this questionnaire demonstrates its applicability in Iranian COVID-19 patients.

Among 13 theoretically and empirically emerged models of the GHQ-12 tested in this study, the current data fitted better with the three-factor model of del Pilar Sánchez-López and Dresch (2008), including successful coping, self-esteem, and stress. The factor loading of all items was adequate (Ford et al., 1986). This result is contrary to Liang et al. (2016) study that showed equal model fit (CFI = 0.98, RMSEA = 0.03) for 11 previously emerged factorial models. The unidimensional model (Goldberg et al., 1997) was not supported in our study, suggesting that GHQ-12 may not be a homogeneous tool that measures only one construct of mental health, or rather, it may cover several constructs instead of concentrating on “narrow aspects” of mental health (Gustafsson and Åberg-Bengtsson, 2010). The established factorial model manifested good reliabilities. Indeed, the overall results of the *Cronbach*’s alpha, *Theta,* and *Omega* coefficients were satisfactory, with the adequate means of inter-item correlations for subscales. These results are in line with a bulk of studies on the psychometric features of GHQ-12 in different contexts (Liang et al., 2016; Elovanio et al., 2020).

To test how accurately the GHQ-12 can correlate the expected outcomes, the criterion validity was conducted as our third objective through the relationship of GHQ with perceived stress, sleep quality, ADL/IADL, and demographic and medical variables. First, GHQ-12 total score and subscales showed significant weak to strong correlations with the sleep quality total score and all subscales, where the subscale of subjective sleep quality had the strongest correlation coefficients. These findings are supported by previous research (Xiong et al., 2019; Aquil et al., 2021; Thielmann et al., 2021). Oh et al. (2019), for instance, found that adults with higher psychological distress had higher difficulty falling asleep. Second, perceived stress showed moderate to high positive correlations with the GHQ-12 total score and three subscales, further supporting the criterion validity of GHQ-12. These findings were consistent with previous studies (Örücü and Demir, 2009; Gajula et al., 2021). It is thought that psychosocial stressors, such as living alone, social restrictions and isolation, financial burden, and loss of family members, that lead to heightened anxiety, fear, and anger, made a significant contribution to the higher level of stress experienced by patients with COVID-19 (Torales et al., 2020; Matalon et al., 2021; Varman et al., 2022).

The GHQ-12 in patients over 60 demonstrated negative correlations with ADL and IADL-as the third criterion variable. A similar result was found by earlier studies (Albanese et al., 2020). Our finding suggested that lack of autonomy in daily life can seriously damage a person’s self-esteem, increase their conflicts with others, and make them more vulnerable to symptoms of anxiety and depression.

The forth variable used to evaluate the criterion validity of GHQ-12 was medical variables, that manifested a set of significant associations. This questionnaire had significant positive correlation with HLP, in line with the study of Wang et al. (2016) that found the GHQ-12 scores was significantly higher in patients with HLP. This association seems bidirectional, given that on the one hand, the empirical evidence suggested that HLP triggers the onset of depression (Chuang et al., 2014), and on the other hand, patients with depression experience a higher incidence of HLP, compared to the general population (Chien et al., 2013). Chang et al. (2021) also showed positive correlation of high blood fat and stress. Kidney failure was shown to be positively correlated with GHQ-12, consistent with two systematic reviews that found a high rate of depression in patients with Kidney failure (Bautovich et al., 2014; Kondo et al., 2020). One explanation for this link might be the impact of difficulties the patients with kidney failure face, such as the psychological and social burden of the disease, comorbid diseases, and the experience of dialysis, which may lead to depression/anxiety (Ozcan et al., 2015). These symptoms are, in turn, associated with negative outcomes including poor quality of life, poor treatment compliance, and elevated mortality rates (Bautovich et al., 2014; Butt et al., 2022). Furthermore, GHQ-12 was significantly correlated with psychiatry disorders. This association is expected because mental illnesses decrease the quality of life and severely impair patients’ ability to communicate and form social relationships. Therefore, it is likely that they are more affected by a pandemic than those with no psychiatric conditions (Kaufman et al., 2020). GHQ-12 was positively correlated with the hospitalization duration. This is another expected result, because at hospitals, patients experience a loss of dignity as a result of their physical conditions, elevating their senses of powerlessness, embarrassment, and being violated. Consequently, these debilitating experiences may lead to mental distress (Liao et al., 2020). Finally, GHQ-12 showed positive correlations with change in sleep time and use of sleeping pills. The change in the sleep–wake cycle might be explained by a third mechanism like anxiety symptoms (Becker et al., 2018). Tang et al. (2017) suggested that higher score of GHQ-12 was strongly predicted by the reduced sleep duration and use of sleeping pills, and vice versa. They argued that extremely long or short sleep duration and excessive use of sleeping pills lead to difficulty in daytime function, which result in adverse outcomes. However, it should be noted that all of these correlations in the current study were weak to moderate and should be interpreted cautiously.

Finally, the criterion validity of GHQ-12 was examined *via* its link with demographic characteristics. GHQ-12 showed negative correlations with educational level and the number of family members. Consistent with our finding, Dalgard et al. (2007) study have shown a significant association between lower educational level and psychological distress in both Norwegian males and female. Additionally, people who live alone may be especially dependent on others for social connection and support, making them more vulnerable to social distancing (Hendriksen et al., 2021). Hence, larger number of family members may be a protective factor against the sense of loneliness and act as a means for social support.

Due to some clues that showed the gender and age differences in the level of GHQ-12, we investigated these group mean differences to be considered in the future use of the questionnaire. In the present study, consistent with previous evidence (Giorgi et al., 2014), lower average of general health in women (higher GHQ-12 total scores and subscales) was observed. Previous evidence have shown that the prevalence of factors thought to be intensified during a pandemic (such as preceding anxiety and depression, chronic environmental exposure, and domestic violence) is higher among women (Bucciarelli et al., 2021). This could increase women’s odds of developing mental health issues. As for age differences, higher level of the GHQ-12 total score and successful coping subscale in patients older than 60 years was found in the present study. This result contradicted previous findings that indicated the association of aging with an intrinsic reduction in susceptibility to psychological distress (Hoeymans et al., 2004). However, losing social contacts in the elderly (e.g., the death of family members), becoming prohibited from engagement in common social interactions due to social distancing order, and receiving limited access to social support and services may increase their susceptibility to mental health problems (Stuart et al., 2022).

### Limitations, future directions, and clinical implications

The current study results provide insight into the general mental health status in patients with COVID-19. However, this study is not without limitations. First, GHQ-12 is a screening tool and was not designed for diagnosis objectives and distinguishing among mental disorders (Goldberg, 1986; Schrnitz et al., 1999). Researchers in future work can use semi-structured interviews to provide more in-depth information regarding high scored items of GHQ-12 and compare yielded scores of GHQ-12 with the additional probe questions (i.e., regarding symptom severity and duration) to evaluate the accuracy of GHQ-12. Second, the cross-sectional design of the current study has prevented causal inferences. It restricts our knowledge on the direction of the correlation of GHQ-12 with perceived stress and sleep quality. It also prevents us from measuring the stability of mental health scores over time. Longitudinal studies are recommended to explore the predictive role of the abovementioned variables on each other, and evaluate the stability of GHQ-12 scores over time. Finally, the present study did not perform the measurement invariance analyses across gender or age, because the sample sizes would start to become small when we divided the sample into age and gender subgroups. In the age case, sample size would be down to 87 for the younger segment. Such small sample sizes lacked sufficient power to detect any invariance.

Given the positive link of poor mental health with perceived stress, sleep disturbances, and impaired independent daily activities among Iranian COVID-19 patients, an important clinical implication for physicians, psychologists, and psychiatrists is to design psychological interventional courses for COVID-19 hospitalized patients that specifically target these problems. Access to such services *via* social media may be beneficial not only for their mental health, but also for their ability to improve their physical and mental functioning and independency (Shojaei and Masoumi, 2020).

## Conclusion

The current study was undertaken to evaluate the psychometric properties of GHQ-12 among Iranian COVID-19 patients. The results of construct validity analyses supported the three-factor model of successful coping, self-esteem, and stress, which showed satisfactory reliability. The criterion validity of GHQ-12 was confirmed through its positive relationship with perceived stress and sleep quality, as well as its negative relationships with activities and instrumental activities of daily living in patients with over 60 years of age. Women and patients above 60 manifested higher GHQ-12 scores, compared to men and patients under 60.

## Data availability statement

The raw data supporting the conclusions of this article can be provided by the corresponding author upon reasonable request.

## Ethics statement

The studies involving human participants were reviewed and approved by the Review Board of Tehran University of Medical Sciences (ethical code: IR.TUMS.VCR.REC.1399.156). The patients/participants provided their written informed consent to participate in this study.

## Author contributions

MHA, FE, and ZV: conceptualization, design, methodology, and investigation and project administration. FF and FG: data collection. MHA: formal analysis and supervision. PSY, NAS, and MHA: writing the original draft. PSY, NW, and MHA: revising the draft. All authors have contributed to the conception and design of the study, drafted or revised this manuscript, reviewed the final version of this manuscript before submission, and agreed to be accountable for all aspects of the work.

## Funding

This research was supported by grant number 99-1-101-47,395 from the Tehran University of Medical sciences.

## Conflict of interest

The authors declare that the research was conducted in the absence of any commercial or financial relationships that could be construed as a potential conflict of interest.

## Publisher’s note

All claims expressed in this article are solely those of the authors and do not necessarily represent those of their affiliated organizations, or those of the publisher, the editors and the reviewers. Any product that may be evaluated in this article, or claim that may be made by its manufacturer, is not guaranteed or endorsed by the publisher.

## Abbreviations

GHQ: General Health Questionnaire
PSS: Perceived Stress Scale
PSQI: Pittsburgh Sleep Quality Index
ADL: activities of daily life
IADL: instrumental activities of daily living
HTN: high blood pressure
HLP: hyperlipidemia
CVA: cerebrovascular accident
HPA: hypothalamic pituitary adrenal
PD: pulmonary disease
IDD: immune deficiency disease
MI: myocardial infarction
CVD: cardiovascular disease
