# Corrigendum: Construct validity of the General Health Questionnaire (GHQ-12) in patients with COVID-19 and its demographic and medical correlates

**DOI:** 10.3389/fpsyg.2023.1333704

**Published:** 2023-12-12

**Authors:** Mojtaba Habibi Asgarabad, Farnaz Etesam, Pardis Salehi Yegaei, Zahra Vahabi, Niusha Akbari Saneh, Fatemeh Fathi, Fatemeh Ghosi, Nora Wiium

**Affiliations:** ^1^Health Promotion Research Center, Iran University of Medical Science, Tehran, Iran; ^2^Department of Psychology, Norwegian University of Science and Technology, Trondheim, Norway; ^3^Department of Health Psychology, School of Behavioral Sciences and Mental Health, Tehran Psychiatric Institute, Iran University of Medical Sciences, Tehran, Iran; ^4^Positive Youth Development Lab, Human Development and Family Sciences, Texas Tech University, Lubbock, TX, United States; ^5^Center of Excellence in Cognitive Neuropsychology, Institute for Cognitive and Brain Sciences, Shahid Beheshti University, Tehran, Iran; ^6^Department of Geriatric Medicine, Tehran University of Medical Sciences, Ziaeian Hospital, Tehran, Iran; ^7^Psychosomatic Research Center, Department of Psychiatry, Tehran University of Medical Sciences, Imam Khomeini Hospital, Tehran, Iran; ^8^Cognitive Neurology and Neuropsychiatry Division, Department of Psychiatry, Tehran University of Medical Sciences, Roozbeh Hospital, Tehran, Iran; ^9^Department of Clinical Psychology, School of Behavioral Sciences and Mental Health, Tehran Psychiatric Institute, Iran University of Medical Sciences, Tehran, Iran; ^10^Faculty of Psychology and Educational Sciences, Semnan University, Semnan, Iran; ^11^Department of Psychology and Educational Sciences, University of Al Zahra, Tehran, Iran; ^12^Department of Psychosocial Science, Faculty of Psychology, University of Bergen, Bergen, Norway

**Keywords:** COVID-19, concurrent validity, general health, medical condition, reliability, sleep, activities of daily life, stress

In the published article, there was an error in the article title. An old version of the title was used. Instead of “Measurement invariance of the General Health Questionnaire (GHQ-12) across gender and age: demographic and medical correlates of mental health in patients with COVID-19”, it should be “Construct validity of the General Health Questionnaire (GHQ-12) in patients with COVID-19 and its demographic and medical correlates.”

In addition, there was an error in Discussion, Paragraph 1. The paragraph included information related to statistical analysis which was removed from our study. The paragraph previously stated: “The present study sought to evaluate the psychometric properties of the General Health Questionnaire-12 in patients hospitalized with the diagnosis of COVID-19. Overall, our results provided support for the construct and criterion validity, internal consistency, and invariance of GHQ-12 across age (under 60 vs. over 60) and gender (male vs. female). Therefore, this questionnaire showed that it can be applied in Iranian COVID-19 patients.” The corrected paragraph appears below:

“The present study aimed to assess the psychometric properties of the General Health Questionnaire-12 in patients hospitalized with a diagnosis of COVID-19. Overall, our results offer support for the construct validity, criterion validity, and internal consistency of GHQ-12. Therefore, this questionnaire demonstrates its applicability in Iranian COVID-19 patients.”

The authors apologize for these errors and state that this does not change the scientific conclusions of the article in any way. The original article has been updated.

