# Supplementary material for: Construct validity of the General Health Questionnaire (GHQ-12) in patients with COVID-19 and its demographic and medical correlates
Source: Front Psychol. 2023 Jun 5;14:1132154. doi: 10.3389/fpsyg.2023.1132154 (PMC10277699; doi:10.3389/fpsyg.2023.1132154)
Supplement: Supplementary file 1 [file Table_1.docx]

**Supporting Information**

**Figure S1.**

Confirmatory factor analysis of the oblique unidimensional model of GHQ-12 (M_1_)


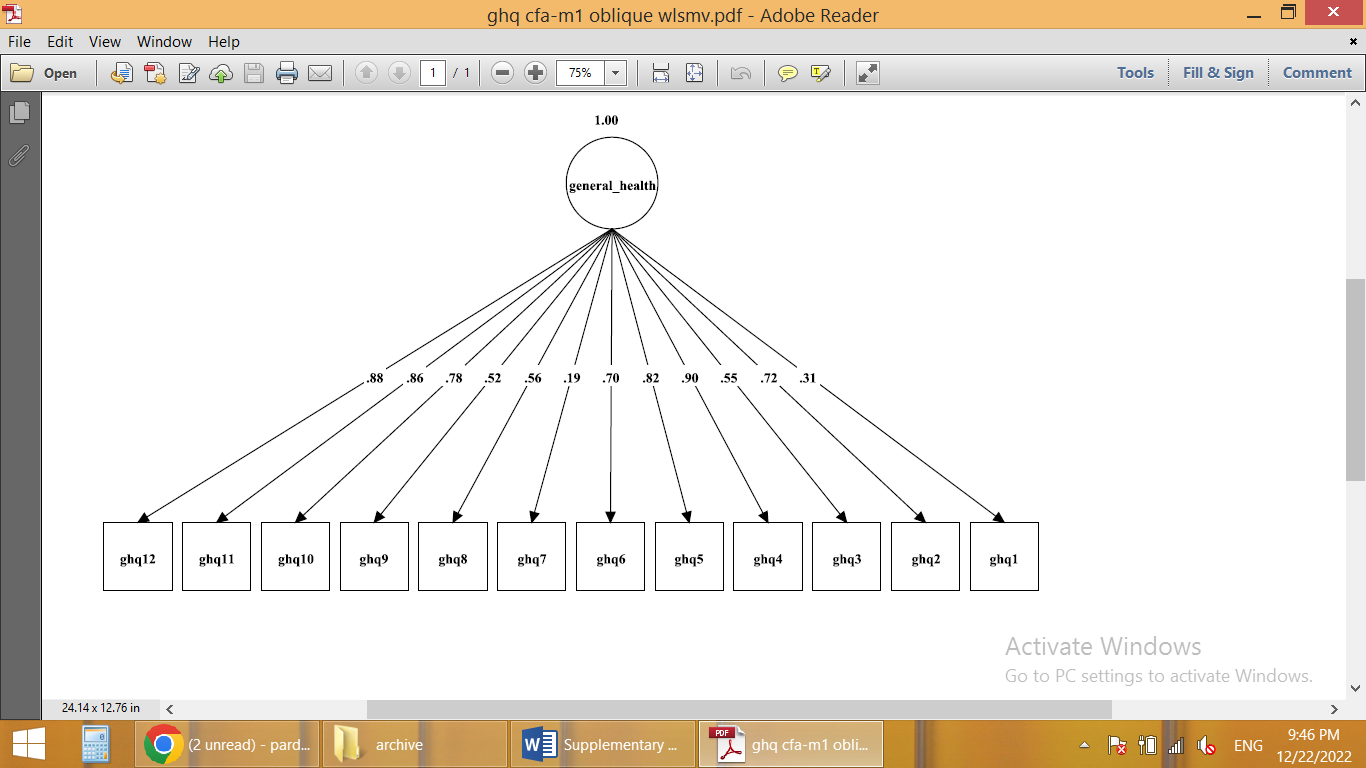


**Figure S2.**

Confirmatory factor analysis of the three-factor oblique model of GHQ-12 (M_8_; Daradkeh et al., 2001)


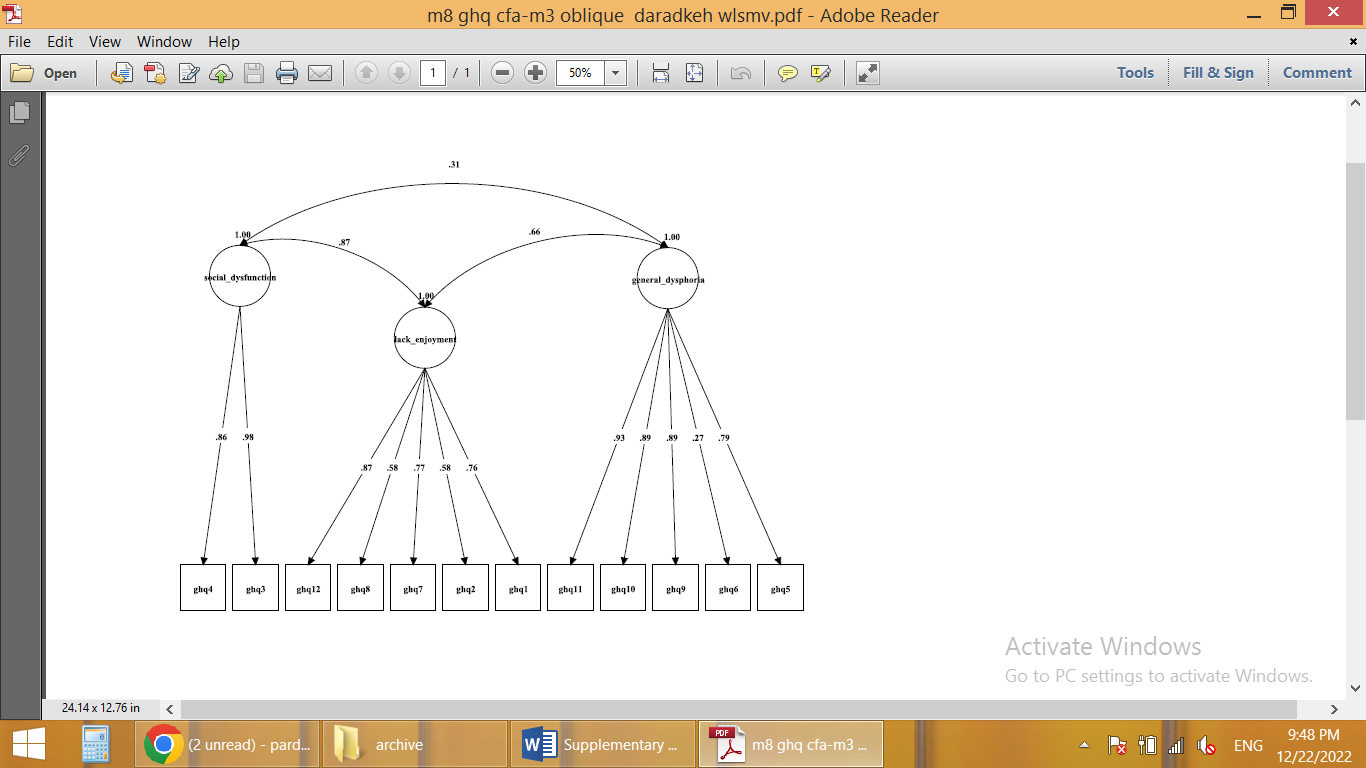


**Figure S3.**

Confirmatory factor analysis of the three-factor oblique model of GHQ-12 (M_10_; Graetz, 1991; Gao et al., 2004; Lee and Kim, 2020)


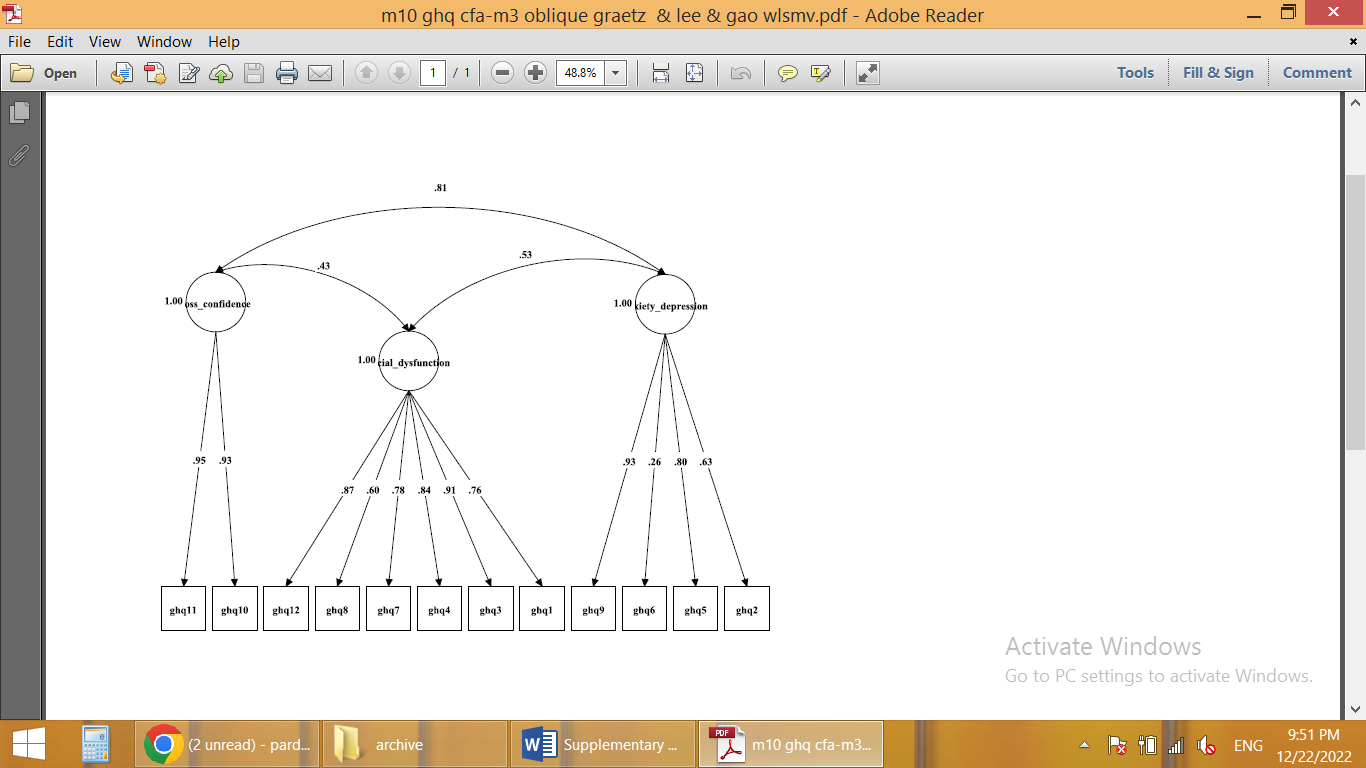


**Figure S4.**

Confirmatory factor analysis of the three-factor oblique model of GHQ-12 (M_11_; Martin, 1999)


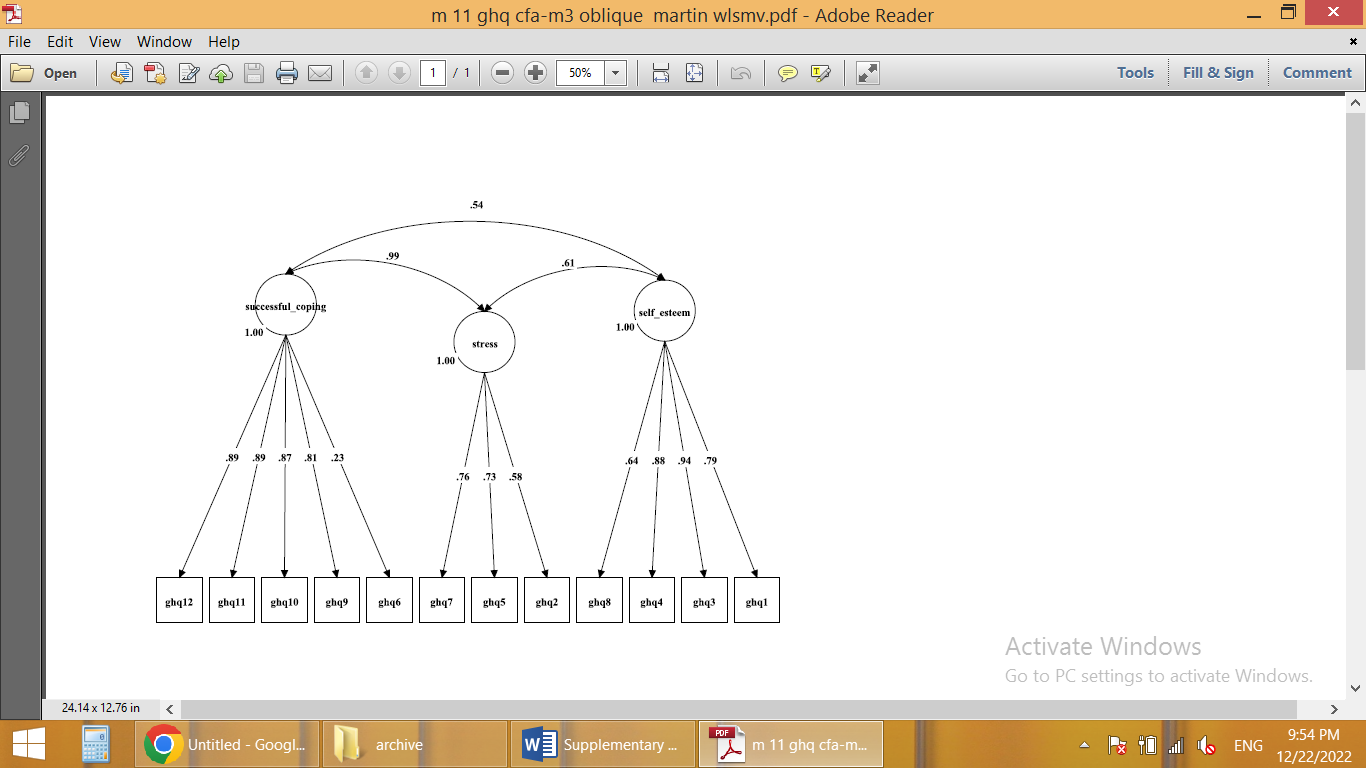


**Figure S5.**

Confirmatory factor analysis of the three-factor oblique model of GHQ-12 (M_13_; del Pilar Sánchez-López and Dresch, 2008)


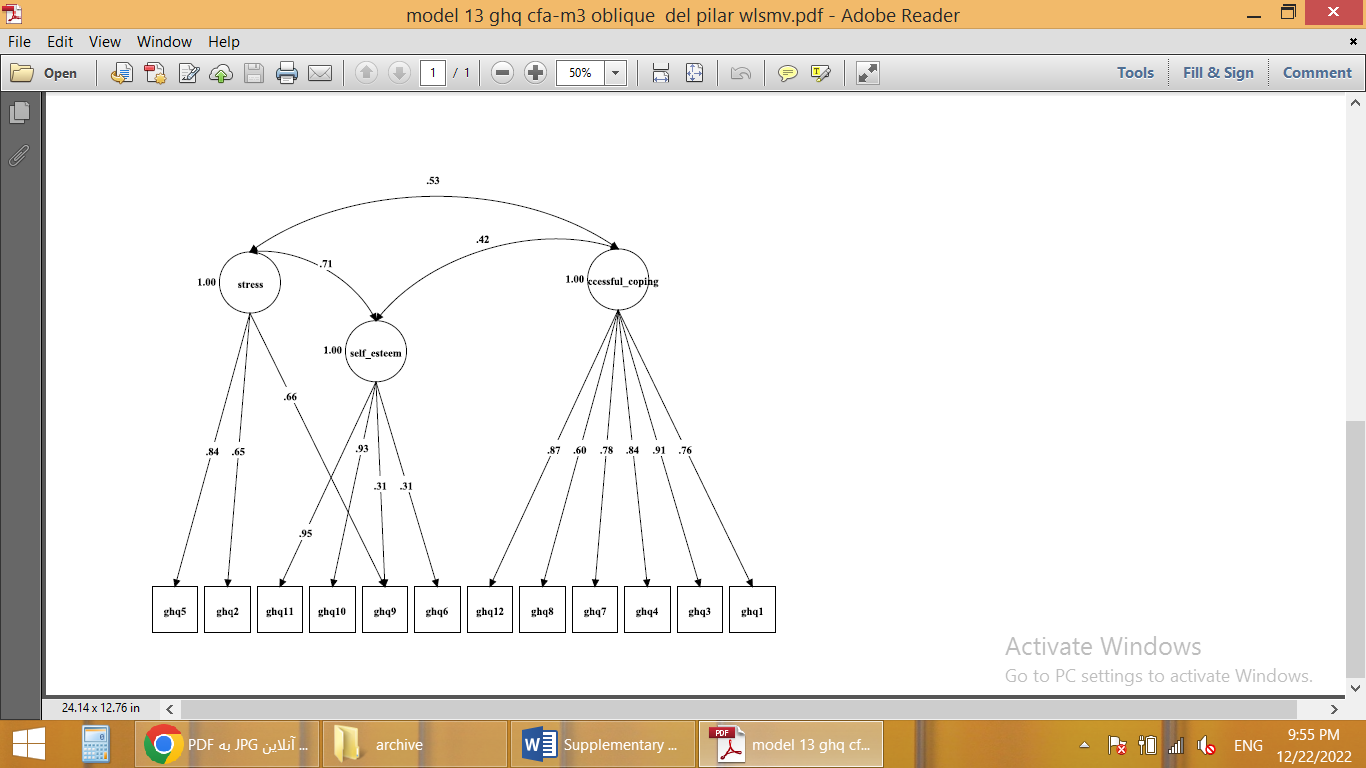


**References**

Daradkeh, T.K., Ghubash, R., and El-Rufaie, O.E. (2001). Reliability, validity, and factor structure of the Arabic version of the 12-item General Health Questionnaire. *Psychological reports* 89(1)**,** 85-94. doi: <https://doi.org/10.2466/pr0.2001.89.1.85>.

del Pilar Sánchez-López, M., and Dresch, V. (2008). The 12-Item General Health Questionnaire (GHQ-12): reliability, external validity and factor structure in the Spanish population. *Psicothema* 20(4)**,** 839-843.

Gao, F., Luo, N., Thumboo, J., Fones, C., Li, S.-C., and Cheung, Y.-B. (2004). Does the 12-item General Health Questionnaire contain multiple factors and do we need them? *Health and Quality of Life Outcomes* 2(1)**,** 1-7. doi: <https://doi.org/10.1186/1477-7525-2-63>.

Graetz, B. (1991). Multidimensional properties of the general health questionnaire. *Social psychiatry and psychiatric epidemiology* 26(3)**,** 132-138. doi: <https://doi.org/10.1007/bf00782952>.

Lee, and Kim, Y.E. (2020). Factor structure of the 12-item general health questionnaire (GHQ-12) among Korean university students. *Psychiatry Clin. Psychopharmacol* 30**,** 248-253. doi: <https://doi.org/10.5455/pcp.20200518112914>.

Martin, A.J. (1999). Assessing the multidimensionality of the 12-item General Health Questionnaire. *Psychological reports* 84(3)**,** 927-935. doi: <https://doi.org/10.2466/pr0.1999.84.3.927>.
